# Rheumatoid arthritis and the intestinal microbiome: probiotics as a potential therapy

**DOI:** 10.3389/fimmu.2024.1331486

**Published:** 2024-03-06

**Authors:** Yang Yang, Qing Hong, Xuehong Zhang, Zhenmin Liu

**Affiliations:** ^1^ State Key Laboratory of Dairy Biotechnology, Shanghai Engineering Research Center of Dairy Biotechnology, Dairy Research Institute, Bright Dairy & Food Co., Ltd., Shanghai, China; ^2^ State Key Laboratory of Microbial Metabolism, and School of Life Sciences and Biotechnology, Shanghai Jiao Tong University, Shanghai, China

**Keywords:** rheumatoid arthritis, gut microbiota, immune response, potential mechanism, probiotics therapy

## Abstract

Rheumatoid arthritis (RA) is a systemic autoimmune disorder characterized by swollen joints, discomfort, stiffness, osteoporosis, and reduced functionality. Genetics, smoking, dust inhalation, high BMI, and hormonal and gut microbiota dysbiosis are all likely causes of the onset or development of RA, but the underlying mechanism remains unknown. Compared to healthy controls, patients with RA have a significantly different composition of gut microbiota. It is well known that the human gut microbiota plays a key role in the initiation, maintenance, and operation of the host immune system. Gut microbiota dysbiosis has local or systematic adverse effects on the host immune system, resulting in host susceptibility to various diseases, including RA. Studies on the intestinal microbiota modulation and immunomodulatory properties of probiotics have been reported, in order to identify their potential possibility in prevention and disease activity control of RA. This review summarized current studies on the role and potential mechanisms of gut microbiota in the development and progression of RA, as well as the preventative and therapeutic effects and potential mechanisms of probiotics on RA. Additionally, we proposed the challenges and difficulties in the application of probiotics in RA, providing the direction for the research and application of probiotics in the prevention of RA.

## 1 Introduction

Rheumatoid arthritis (RA) is a chronic autoimmune disease that can lead to joint swelling, joint pain, morning stiffness, joint deformities, osteoporosis, and even disability (1). RA affects approximately 0.5-1.0% of adults aged 20-40 years globally and is more prevalent among individuals over the age of 75 (2, 3). Due to the unclear etiology and pathogenesis of RA, the main objective of treatment is to reduce joint inflammation and control the progression of lesions. The drugs used to treat RA include conventional synthetic disease-modifying antirheumatic drugs (csDMARDs), biologic DMARDs (bDMARDs), targeted synthetic DMARDs (tsDMARDs), and glucocorticoids (4). Early treatment is essential in RA management as it can effectively prevent irreversible structural damage and chronic functional impairment (5). Prior to the onset of clinical symptoms, RA is preceded by a preclinical phase that can last several years, during which RA-related autoimmunity is serologically detectable. This phase is characterized by the presence of autoantibodies, namely anti-citrullinated protein antibodies (ACPAs) and Fc domain-recognizing rheumatoid factors (RFs) (6). As RA progresses, synovial inflammation develops due to the infiltration of mononuclear cells, particularly CD4^+^ T cells, and macrophages, along with early activation of stromal cells. Inflammatory cells accumulate in the synovium, leading to the formation of a pannus that can destroy cartilage and further propagate the inflammatory process, eventually leading to clinical arthritis (7).

The symptoms of RA are associated with inflammation. Two key pathogenetic changes in the synovium in RA are an increase and activation of synoviocytes (8), as well as the infiltration of adaptive immune cells into the synovial sublining (9). These alterations are a prominent source of cytokines, proteases, and antibodies. The inflammation in the synovium leads to synovial hyperplasia and invasion into adjacent articular structures and eventually joint damage (10). The pathways involved in joint damage may include the release of cytokines and matrix metalloproteinases (MMPs) of macrophages, neutrophils (particularly in the synovial fluid) and mast cells (7). The maturation and activation of osteoclasts are the dominant factors of bone damage, while the interaction between ACPAs and citrullinated peptides expressed by osteoclasts and osteoclast precursors was the reason for osteoclast maturation and activation (11). However, studies suggest that the interaction above could precede the onset of synovial inflammation (12, 13).

A variety of risk factors are involved in the development of RA, including genetic and environmental factors. Studies on genetic predisposition to RA have implicated RA-related genes such as human leukocyte antigen (HLA) alleles as well as many other genes with low relative risk variants. Specific class II HLA loci can encode major histocompatibility complex (MHC) molecules that may contain the shared epitope (SE) (14). SE is a specific amino acid motif and is characterized by conserved amino acids within the peptide-binding groove, which are linked to an increased risk of developing RA (15). Moreover, environmental factors such as smoking, dust inhalation, high BMI, and hormonal factors have been demonstrated to contribute to the pathogenesis of RA (16). Heavy smoking, for example, heightens the likelihood of testing positive for ACPAs that are associated with joint symptoms (17, 18). Exposure to silica is also correlated with an elevated risk of future RA development (19).

In addition, various studies have suggested that mucosal environmental exposures and/or mucosal dysbiosis play a causal role in the period of susceptibility to RA (20–23). These researches support the ‘mucosal origins hypothesis’, which says that RA originates at one or more mucosal sites and then is transmitted to other organs. However, the hypothesis is not able to identify as to what stage of the development of RA this mucosal origination occurs. It is known that the gastrointestinal tract is the largest mucosal barrier, and is an essential getaway for the host to take in nutrition from food and drinks (24, 25). Up to 100 trillion symbiotic microbes live in the gut, and these microbes are involved in the digestion, metabolism, nutrition, disease control, and maintenance of general well-being (26, 27). The imbalance of gastrointestinal homeostasis, especially dysbiosis in gut microbiota composition and diversity, results in inflammatory responses and further triggers the occurrence of diseases such as RA (28, 29). Probiotics are a potential adjuvant to restore their original intestinal balance and behavior, and to modulate both innate and adaptive immunity in the host. The use of probiotics prevents or ameliorates the early stage of disease, and may be an effective therapy for chronic inflammatory diseases (30).

In this review, we summarize recent literature to help better understand the role of gut microbiome in RA, and to evaluate the therapeutic and preventative effects of probiotics in RA, providing a solid framework for future studies of optimizing the management of the health status of patients with RA.

## 2 Crosstalk among RA, gut microbiota, and immune system

**Figure 1:**
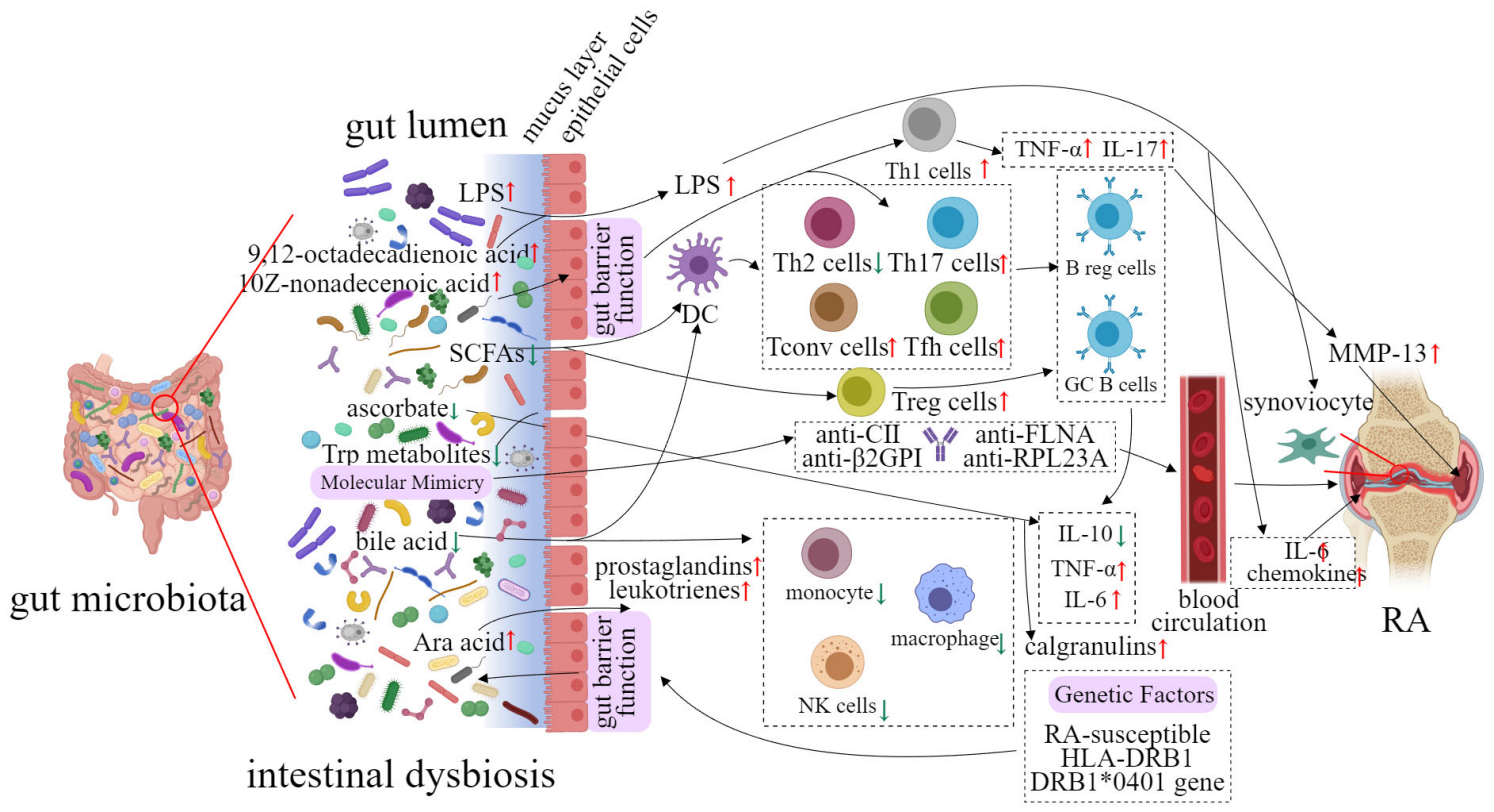
Potential implication of gut microbiota in the etiology of RA.

The gut microbiota has gained more and more accentuated attention from specialists as a key contributor to the development and progression of RA (31, 32). In a study, fecal samples from RA patients were transplanted to germ-free mice, the result showed that mice inoculated microbiota from RA patients had an increased level of Th17 cells in the gut and an increased risk of developing severe arthritis (33). With continuous research, it has been found that perturbations of gut microbiota have been observed at different stages of RA, such as pre-clinical RA and established RA. In addition, these perturbations of gut microbiota are able to affect the host`s immune response (**Figure 1**).

### 2.1 Pre-clinical RA and gut microbiota

Rogier and his colleagues reported that the gut microbiota had a significant alteration in the pre-clinical stage of collagen-induced arthritis (CIA) mice (34). In pre-clinical RA mice, the abundance of the phylum *Bacteroidete* (e.g. S24-7 and *Bacteroidaceae*) was decreased, while *Firmicutes* and *Proteobacteria* (e.g. *Ruminococcaceae*, *Lachnospiraceae*, and *Desulfovibrinocaceae*) were increased (34). Interleukin (IL) 17-producing T helper 17 cell counts and the severity of RA was reduced when the gut microbiome was eliminated in established RA mice (34). In a study on anti-citrullinated protein (anti-CCP) positive individuals, researchers have sequenced the 16S rRNA gene of their gut microbiota (35). The abundance of *Lachnospiraceae*, *Helicobacteraceae*, *Ruminococcaceae*, *Erysipelotrichaceae*, and *Bifidobacteriaceae* were significantly increased in the intestine of anti-CCP-positive individuals (35). Furthermore, five out of twenty-five anti-CCP-positive individuals have progressed to RA during this trial (35).

### 2.2 Established RA and gut microbiota

A clinical trial involving 32 patients with RA and 30 healthy controls revealed significant differences in intestinal bacteria composition. Lactobacteria were found to be significantly decreased in RA patients (*P* < 0.05), whereas *Enterococci* and *Clostridia* were significantly increased (*P* < 0.05). In addition, the proportion of *Bifidobacteria*, *Bacteroids*, and *Lactopositive colibacteria* was observed to be reduced (*P* < 0.05), while the abundance of opportunistic *Enterobacteria* and *Staphylococci* showed increased (*P* < 0.05) (36). It is worth noting that opportunistic *Enterobacteriaceae* in urine and nasal mucosa were also detected (*P* < 0.05). This result implied the possible fact that opportunistic *Enterobacteriaceae* translocated from the intestines to other organs (36). In another cohort of individuals with RA and healthy subjects (at the age of 18-65), Zhang and his colleagues analyzed the change of microbiome in fecal, dental, and salivary samples respectively from RA patients through metagenomic sequencing and metagenome-wide association study (22). The results showed that *Haemophilus* spp. were depleted, while *Lactobacillus salivarius* was over-represented in the gut of RA patients (*P* < 0.05, Z-score≥1.6, 90% confidence). Similar findings were observed in their dental and salivary samples (22).

### 2.3 Gut microbiota and immune system

The gut microbiota plays an essential role in the maturation and development of the gut-associated lymphoid tissue (GALT) (37). GALT is composed of separate or aggregated lymphoid follicles, which form Peyer’s patches (PPs). PPs are considered to be an immune sensor in the gut, capable of controlling antigens and bacteria in the lumen area (38). Moreover, the interaction between follicle-associated epithelium and immune cells in PPs is associated with immune defense and immunologic tolerance (38). The lack of gut microbiota leads to a decreased level of mesenteric lymph nodes (MLNs) and PPs, as well as a reduced number of immune cells (39, 40). However, when gut microbiota is colonized in the intestines, the immune system can be rebuilt (41).

*Faecalibacterium prausnitzii* is one of the important commensal bacteria in the human gut microbiota, accounting for 3-5% of the total number of bacteria detected in stool samples of healthy people (42). A decrease in the abundance of *F. prausnitzii* in the intestine may be a sign of dysregulation such as the reduced capacity of self-defense against inflammatory reactions (43, 44). Furthermore, metabolites produced by the intestinal flora also influence intestinal immunity. *Bacteroidetes fragilis*, a symbiotic bacterium in humans, belongs to the phylum *Bacteroidetes*, which is one of the four major phyla of intestinal bacteria viz. *Bacteroidetes*, *Firmicutes*, *Proteobacteria*, and *Actinobacteria*. Polysaccharide A, secreted by *Bacteroides fragilis*, induces CD4^+^ T cells to transform into Foxp3^+^ regulatory T cells (Tregs) that produce IL-10 (45). Toll-like receptor 2 signaling is active by Tregs and IL-10 (45).

The above studies have demonstrated that the intestinal commensal microbiota is able to regulate T cell and Treg responses thereby protecting the host from pathogens. Intestinal commensal bacteria are crucial for the establishment of a regular innate immune system (46). As the human body’s first line of defense, innate immune responses rely on a family of receptors named pattern recognition receptors (PRRs). PRRs, especially Toll-like receptors (TLRs), are able to recognize pathogen-associated molecular patterns (PAMPs), and play a vital role in immune defense (47). TLRs are recognized by both gram-positive and gram-negative bacteria to regulate innate and adaptive immunity (48). Gram-positive bacteria, gram-negative bacteria, and their related PAMPs are identified by TLR-2 and TLR-4, respectively (49). TLR-2 pathway is activated by peptidoglycans and polysaccharides of gram-positive bacteria`s cytoderm. TLR-4 is bound to lipopolysaccharide of gram-negative bacteria (50). However, the imbalance of gut microbiota is likely to cause the alteration of the innate immune system. These alterations allow microbes and their metabolites to cross into the lamina propria and sub-epithelial spaces, which is related to the risk and severity of the disease such as RA (51).

## 3 Potential mechanisms linking gut microbiota to RA

Many studies suggest a potential link between RA development and intestinal dysbiosis (46, 52–56). Recent clinical studies have demonstrated that alterations in gut microbiota and their metabolite concentrations occurred before RA onset (57–61). These alterations activate autoreactive T cells and may induce systemic inflammation (33). The potential mechanism linking gut microbiota to RA will be elaborated below.

### 3.1 Molecular mimicry

The mechanism of molecular mimicry has been known for a long time (62), but only in recent years have we begun to focus on the molecular mimicry of microbes that coexist with humans. Studies have revealed that human microorganisms express and produce some homologous proteins similar to host proteins, and these proteins may cause host immune imbalance under some circumstances, resulting in the development of autoimmune diseases (63–65).

Citrullination is a normal physiological process, involved in various physiological functions such as cell apoptosis, end-stage differentiation, gene regulation, and reproductive development. Peptidyl arginine deiminase (PAD) catalyzes the conversion of peptidyl arginine to citrulline in the involvement of Ca^2+^ (66). However, PAD secreted by *Porphyromonas gingivalis* (PPAD) enables catalyzed citrullination without Ca^2+^ (67). *Porphyromonas gingivalis* is the main cause of periodontal disease, but it is also highly correlated with RA (68). Patients with periodontal disease have a two-fold increased risk of RA (69). Severity of periodontitis is related to the severity of RA (70). PPAD can catalyze the citrullination of not only *P. gingivalis*`s proteins but also a variety of proteins such as vimentin, fibrinogen, and α-enolase, which are RA-specific autoantigens. Further studies also found that anti-CCP proteins were detectable in RA patients (71). The presence of citrullinated proteins was observed in the synovium of other inflammatory arthritis such as osteoarthritis, but there is no anti-CCP (72). These results suggested that citrullinated proteins may not be specific for RA, but the immune response against citrullinated proteins is unique to RA. This further illustrates that recognition of citrullinated proteins by autoreactive T cells and production of ACPAs by B cells may be even more important for RA. In addition to *P. gingivalis*, the others are also closely related to molecular mimicry and RA (**Table 1**).

**Table 1:** Molecular mimicry of microbiome in RA.

Microbiome	Immune response	Stage of RA	Ref.
*Bacteroidaceae*, *Lachnospiraceae*, and S24-7	The higher level of the cytokine IL-17 in serum and the ratios of CD8^+^ T cells and Th17 lymphocytes in the spleen; The smaller number of dendritic cells, B cells, and Treg cells in the spleen.	The progression of RA	(73)
*Haemophilus* spp., *Lactobacillus salivarius*	Serum anti-CCP, RF, CRP, IgG levels, and DAS28 were increased.	Established RA	(22)
*Prevotella copri*	The number of intestinal Th17 cells was increased; The level of IL-17 in lymphocytes of regional lymph nodes and the colon was enhanced.	early RA	(33)
*Aggregatibacter actinomycetemcomitans*	The hypercitrullination in host neutrophils was induced; The activation of citrullinating enzymes in neutrophils was dysregulated.	Pre-clinical RA	(74)
*Anaeroglobus geminatus*	The presence of ACPAs/rheumatoid factor was observed.	New-onset RA	(75)
*Cryptobacterium curtum*	Large amounts of citrulline were produced.	Established RA	(76)
*Defluviitaleaceae_UCG-011*, *Neisseria oralis*, *Prevotella_6*	Serum ACPAs titers were positive; The immunoglobulin G (IgG), C-reactive protein (CRP), and erythrocyte sedimentation rate (ESR) levels were increased.	Pre-clinical RA	(77)

### 3.2 Shared epitope

Human leukocyte antigen (HLA) genes have been found to be associated with RA (78). It was found that the majority of RA patients share conserved amino acid sequences in the third hypervariable region (HV3) of the HLA DR molecule, which has been termed a ‘‘shared epitope’’ (SE) (14, 79). The SE-coding *HLA-DRB1* alleles are the most significant genetic risk factor associated with RA (80, 81). It is worth noting that a cross-sectional study of 1650 TwinsUK participants showed that *Prevotella spp* was significantly associated with the RA polygenic risk score. And SCREEN-RA participants (n=133) carried established SE risk alleles. These results implied that *Prevotella spp* in the gut microbiota was associated with RA genotype before pre-clinical RA (58).

The mechanism underlying the role of the SE in RA remains unclear. There is a hypothesis that the SE may act as a signal transduction ligand that can trigger innate immune signaling (78). The hypothesis is based on the fact that the SE is located near the apex of α helical tri-dimensional structural motif of the major histocompatibility complex (MHC) gene family. The MHC, a group of genes, encodes major histocompatibility antigens in animals. The human MHC is named HLA. It is known that products coded by the MHC gene family have tri-dimensional homology (82). The top of α helical tri-dimensional structural motif seems to be enriched in signal transduction ligands. Therefore, it is presumed that SE is similar to class I MHC-coded molecules, resulting in stimulating innate immune responses (78). Studies show that SE is bound to the P-domain of calreticulin (CRT, a known innate immunity receptor) on the cell surface (83). The amino acid residues 217-224 of the CRT P-domain are a potential SE binding site (84). Further investigations have revealed that the SE-CRT pathway is involved in the activation of NO synthase and the production of reactive oxygen species (ROS), consequently leading to the inducing osteoclast-mediated bone destruction (85–87).

It is speculated that SE activates immune dysregulation. CRT is expressed on the surface of many cells including dendritic cells (DCs) (88). Apoptotic cells determine whether a pro- or anti-inflammatory reaction is activated in the host, whereas CRT plays a pivotal role in the clearance of apoptotic cells (89). This makes CRT play an important role in the junction between immunologic tolerance and autoimmunity (90). As professional antigen-presenting cells, DCs also induce tolerance mediated partly by indoleamine 2,3-dioxygenase (IDO) which is an enzyme that catalyzes the catabolism of tryptophan (91, 92). Thus, SE activates immune dysregulation by inhibiting the activity of IDO in DCs (93). In addition, a SE ligand stimulates the production of IL-6 and the differentiation of Th17 (94, 95). The above studies have gradually demonstrated that SE could affect RA pathogenesis.

## 4 Probiotics and rheumatoid arthritis

### 4.1 The therapeutic effect of probiotics

The definition of probiotics is “Live microorganisms which when administered in adequate amounts confer a health benefit on the host.” (96). Probiotics exert various effects on the body, with different probiotics functioning in distinct ways. One possible way involves probiotics aiding the host in maintaining a healthy microbiome, as well as assisting in the restoration of intestinal microbial balance after dysbiosis. Additionally, probiotics have the ability to produce bioactive substances that elicit the desired effects and influence the immune responses of the host.

Probiotics have been studied in animal and human trials to assess their potential positive effects on the prevention and treatment of RA (**Table 2**). Probiotics such as *Lactobacillus* and *Bifidobacterium* have been widely investigated. In a study involving rats, different Lactobacillus species were orally administered two weeks before the induction of collagen-induced arthritis (CIA). The results demonstrated that different types of *Lactobacillus* exerted varying effects on RA. *L. reuteri*, *L. casei*, *L. rhamnosus*, and *L. fermentum* were found to alleviate RA by inhibiting species-specific pro-inflammatory cytokine and anti-CII antibody signaling pathways and regulating the balance of microflora and metabolites (e.g., short-chain fatty acids) through species-dependent immune regulation in the gut (106). In particular, *L. reuteri* and *L. casei* had a decreased effect on Th1 immune responses, whereas *L. rhamnosus* and *L. fermentum* had a decreased effect on Th17 immune responses. *L. plantarum* reduced both Th1 and Th17 immune responses, but it did not attenuate RA. Conversely, *L. salivarius* only postponed the onset time of RA without inducing the immune response (106). In a randomized and double-blind clinical trial, 46 RA patients were provided a daily capsule of *L. casei* 01 for 8 weeks. The serum level of pro-inflammatory cytokines (tumor necrosis factor-α, interleukin-6, interleukin-12) was significantly decreased. The serum regulatory cytokine (interleukin-10) was increased. The disease activity and inflammatory status of these participants were significantly decreased by *L. casei* 01 supplementation (107). Similarly, oral administration of *L. rhamnosus* GR-1 and *L. reuteri* RC-14 was found to improve the Health Assessment Questionnaire (HAQ) score of active RA patients and achieved an American College of Rheumatology (ACR) 20 response (108).

**Table 2:** List of human and animal studies on the therapeutic effect of probiotics.

Probiotics	Duration	Study design	Main findings	Ref.
*Lactobacillus casei* ATCC 334	28 days (2 × 10^8^ CFU/ml)	Animal experiment	• The synovial membrane and cartilage were histopathologically normal without bone destruction; • A decrease in arthritis score and pro-inflammatory cytokines.	(97)
*Lactobacillus casei* ATCC 334	30 days (2 × 10^8^ CFU/day)	Animal experiment	• Less joint swelling, lower arthritis scores, and less bone destruction; • A reduction in dysbiosis of the intestinal microbiome.	(98)
*Lactobacillus casei*	12 weeks (4 × 10^10^ CFU/g)	Animal experiment	• The clinical symptoms were reduced, the swelling of the paw, the infiltration of lymphocytes, and the destruction of cartilage tissue were reduced; • An increase in anti-inflammatory cytokines (IL-10 and TGF-β); • A decrease in pro-inflammatory cytokines (IL-1β, IL-2, IL-6, IL-12, IL-17, IFN-γ and TNF-α) • A decrease in CII-reactive T cell proliferation and the levels of Th1-type IgG isotypes (IgG2a and IgG2b); • An up-regulation in the expression levels of Foxp3 and an increased population of Foxp3^+^ CD4^+^ T cells.	(99)
*Lactobacillus plantarum MTCC No. 1047*	21 days (10^5^, 10^7,^ 10^9^ CFU/animal)	• Animal experiment • Complete Freund’s adjuvant (CFA)-induced arthritis in rats	• Inhibition of the release of inflammatory markers like CRP, ESR, RF, and TNF-α. • An improvement in arthritic index and joint stiffness, gait test, and mobility test.	(100)
*Lactobacillus casei* 01	8 weeks (10^8^ CFU/day)	• Randomized doublle-blind clinical trial • 46 RA patients in Iran	• A decrease in SOD activity and GPx activity	(101)
*Lactobacillus casei* 01	8 weeks (10^8^ CFU/day)	• Randomized double-blind clinical trial • 45 RA patients who had disease duration of more than 1 year at the age of 20-80 in Germany	• A decrease in serum hs-CRP levels, tender and swollen joint counts, GH score and DAS28; • A significant difference was observed between *L. casei* 01 and placebo groups for IL-10, IL-12, and TNF-α changes.	(102)
*Lactobacillus rhamnosus* GG (LGG)	12 months (5 × 10^9^ cfu/capsule, 2 capsules/day)	• Double‐blind clinical trial • 21 RA patients who had a disease duration of at least 1 year at the age of 18-64 in Finland	• A decrease in the mean number of tender, swollen joints and RA activity; • A slight increase in serum IL‐1β.	(103)
*Lactobacillus acidophilus*, *Lactobacillus casei* and *Bifidobacterium bifidum*	8 weeks (2 × 10^9^ CFU/g each)	• Randomized, double-blind, placebo-controlled trial • 54 patients with RA at the age of 25-70 in Iran	• A reduction in serum hs-CRP levels, insulin values, HOMA-IR and HOMA-B; • An increase in plasma NO, plasma glutathione, DAS-28, and VAS pain.	(104)
*Lactobacillus acidophilus La-14, Lactobacillus casei Lc-11, Lactococcus lactis Ll-23, Bifidobacterium lactis Bl-04 and B. bifidum Bb-06*	60 days (10^9^ CFU/g each)	• Randomized and double-blind placebo-controlled study • 42 RA patients in Brazil	• A reduction in white blood cell count and TNF-α and IL-6 plasma levels • A reduction in NO metabolites, and an increase in the sulfhydryl group and total radical-trapping antioxidant parameters	(105)

hs-CRP, high-sensitivity C-reactive protein; GH, global health; DAS28, Disease activity score-28; SOD, superoxide dismutase; GPx, glutathione peroxidase; VAS, visual analogue scales; HOMA-IR, homoeostasis model of assessment-estimated insulin resistance; HOMA-B, homoeostatic model assessment-β-cell function.

*Bifidobacterium* species are also often studied for their effects on RA. In an early intervention experiment, five strains of *Bifidobacterium adolescentis* including HuNan2016-7-2, AHWH4-M1, FSDJN3Q1, M1DZ09M1, and FSDJN12W5 were given to CIA rats respectively by gavage. The clinical symptoms of CIA rats were alleviated, and the balance between pro- and anti-inflammatory responses was restored. Furthermore, the dysbiosis in the gut microbiota of the CIA rats was also rectified (109). In a pre-clinical model, intervention with *B. longum* RAPO in CIA mice, obese CIA, and humanized avatar model ameliorated the symptoms of RA such as reduced RA incidence, arthritis score, inflammation, bone damage, and cartilage damage. Additionally, *B. longum* RAPO may play a role in ameliorating RA via inhibiting the secretion of IL-17 and other pro-inflammatory mediators, suggesting its potential role in ameliorating RA (110). Furthermore, three strains containing *L. acidophilus*, *L. casei*, *B. bifidum* had beneficial effects on RA patients. The Disease Activity Score of 28 joints (DAS-28) of RA patients who were given probiotic capsules was improved. The homeostatic model assessment-B of cell function and serum high-sensitivity C-reactive protein concentrations had a significant decrease (111). In a patent, a newly isolated strain called *Bifidobacterium bifidum* ATT was found to be an effective composition for alleviating, preventing, or treating RA (112).

*Prevotella is a newly discovered species that shares taxonomic similarities with Prevotella nigra and Prevotella villi.* In a study by Marietta and colleagues (113), arthritis-susceptible HLA-DQ8 mice were treated with *P. histicola*, resulting in a significant decrease in the incidence and severity of arthritis in DQ8 mice. Moreover, the study observed a suppression of antigen-specific Th17 responses, an increase in the transcription of IL-10, and an upregulation of antimicrobial peptidase and tight junction proteins (113). On the other hand, it is possible that *P. histicola* colonization in the duodenum contributes to the restoration of *Allobaculum* population in the host’s gut. This colonization could potentially lead to an increase in butyrate production, and consequently higher levels of short-chain fatty acids (SCFAs). These elevated levels of SCFAs could potentially contribute to the maintenance of immune homeostasis (114).

*Bacillus* probiotics are a type of probiotic bacteria isolated from the human intestine, with *Bacillus coagulans* being one of the most common strains, known for its ability to produce lactic acid. In a double-blind trial, 45 patients with RA who were administered *B. coagulans* GBI-30, 6086 for 60 days showed a significant improvement in the Patient Pain Assessment score and Pain Scale compared to the placebo group. Moreover, some participants also demonstrated enhanced mobility, as evidenced by their ability to walk 2 miles and engage in daily activities (115). Additionally, *B. coagulans* also generates SCFAs including butyric acid, which is recognized for its role in promoting gut cell health and healing, as well as its involvement in regulating the mucosal immune system (116). In an *in vivo* experiment, arthritic rats were orally administered a combination of probiotic *B. coagulans* and prebiotic inulin to evaluate their possible influence on RA. The results indicated that pretreatment with *B. coagulans* and inulin significantly inhibits the production of serum amyloid A and fibrinogen, as well as the development of paw swelling in arthritic rats. Notably, the combination probiotic group displayed a significant reduction in pro-inflammatory cytokines, such as TNF-α (117).

### 4.2 Potential mechanisms

**Figure 2:**
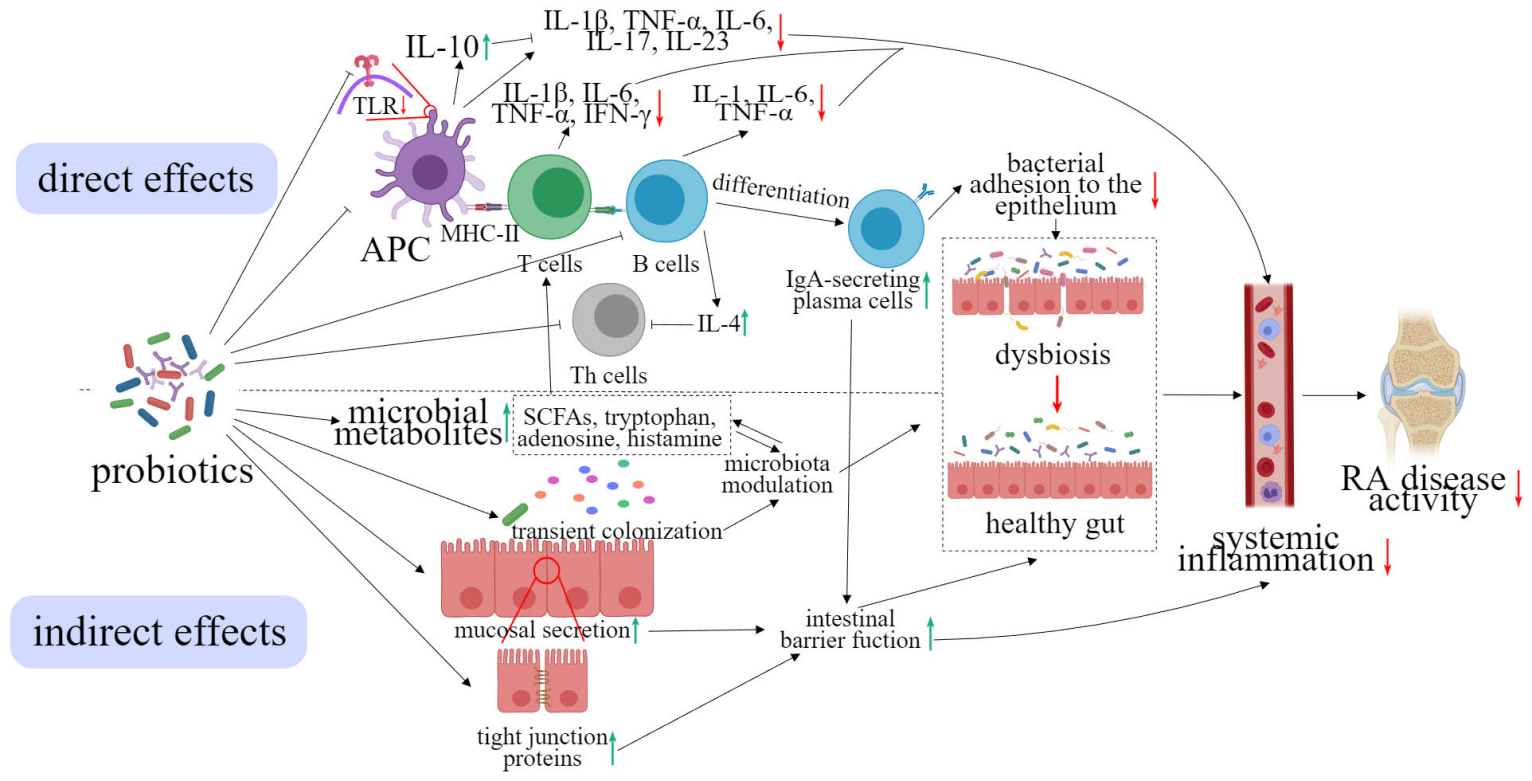
Effect of probiotics on the reduction of the symptoms of RA.

The underlying mechanisms through which probiotics exert their anti-inflammatory and immunomodulatory effects are not yet fully understood. However, it has been observed that probiotics can regulate systemic inflammation through both direct and indirect pathways, ultimately leading to a reduction in RA disease activity (118) (**Figure 2**). In the direct pathway, specific probiotics have been shown to have an impact on various cells associated with innate and acquired immunity, including epithelial cells, macrophages, dendritic cells (DCs), natural killer cells (NKs), T cells, and B cells (119). The expression of PRRs on immune and non-immune cells (such as NK cells, DCs, macrophages, fibroblasts, and epithelial cells) is decreased by specific probiotics. Antigen-presenting cells (APCs) and Th cells are decreased. Th cells are also inhibited by the increase of IL-4 that is secreted by B cells (120). The pro-inflammatory cytokines such as IL-1β, TNF-α, IL-6, IL-17, and IL-23 are suppressed by anti-inflammatory cytokines IL-10 that is produced by APCs, resulting in the decrease of systemic inflammation (121). Furthermore, the increasing differentiation of B cells into IgA-secreting plasma cells leads to a restoration of the intestinal barrier function and a suppression in the bacterial adhesion to the epithelium. In the indirect pathway, probiotics are believed to promote the production of microbial metabolites such as SCFAs, tryptophan, adenosine, and histamine (122). This can lead to competition with pathogenic bacteria, reducing the likelihood of pathogenic microorganisms entering the system. Probiotics also stimulate mucosal secretion and promote the expression of tight junction proteins, further restoring the function of the intestinal barrier (123). Furthermore, microbial metabolites also play a role in regulating gut microbiota and changes in microbiota can impact the production of these microbial metabolites. Overall, these mechanisms contribute to the restoration of the balance of intestinal microbiomes and a reduction in systemic inflammation, ultimately leading to a decrease in RA disease activity (124).

SCFAs are one of the most abundant metabolites of gut microbiota and play a role in modulating the immune system. Specifically, SCFAs have been shown to inhibit the differentiation of DCs (125) and alter the expression of cytokines by DCs (126). Additionally, SCFAs such as butyrate and propionate can induce apoptosis and affect the phagocytic capacity of neutrophils (127). Moreover, butyrate, acetate, and propionate have been found to down-regulation pro-inflammatory mediators in macrophages, that are induced by lipopolysaccharides (LPS) (128, 129). SCFAs also have a positive impact on intestinal barrier function by upregulating the expression of tight junction proteins (130). In addition, butyrate has been shown to suppress the secretion of inflammatory cytokines and inhibit RA through its regulation of Treg cells (131). This connection between SCFAs and their immunomodulatory effects has established their significance in the context of RA.

## 5 Challenges

The etiology and pathogenesis of RA remain unresolved, making the search for highly effective treatment methods, preventive measures, and specific medicine an ongoing area of exploration (132). The primary objective in treating RA is to decrease joint inflammation, hinder the progression of lesions and irreversible bone damage, and safeguard the functionality of joints and muscles. The ultimate goal is to attain complete remission of the disease. Overall, RA exhibits a slow onset and chronic course, which can manifest varying degrees of difficulties and pain in daily life and work (133). However, with early diagnosis and prompt, comprehensive treatment, most patients can achieve varying degrees of effectiveness in alleviating pain and controlling disease progression (4).

Based on previous studies, probiotic treatment can alleviate symptoms of RA, but the underlying mechanism remains unknown. Several possible pathways have been proposed, including molecular mimicry (134), shared epitope (95), intestinal immune responses (6), metabolites produced by the intestinal flora (124), and compromised intestinal barrier function (135) associated with gut microbiota dysbiosis. To validate these pathways further research is needed. For instance, the ‘mucosal origins hypothesis’ says that inflammation is transmitted from one mucosal site to further ones, and it is necessary to investigate whether a single site is sufficient to trigger systemic spread through additional studies. The relationship between local mucosal findings and systemic immune changes should also be further examined (6). Additionally, it is important to evaluate T cell antigen-specific or innate immune reactivity systemically and at mucosal sites. Thus, ongoing research, such as APIPPRA (136) and TREAT EARLIER (137), has extended their observation period to up to 5 years to assess the long-term sustainability of the preventive effects of RA (138, 139). These long-term data will be crucial for a comprehensive understanding of the mechanisms underlying RA development and for reducing the disease burden in at-risk individuals.

A probiotic with optimal performance must undergo a series of processes including isolation, screening, evaluation, and final application, production, and promotion. These processes encompass various research aspects, such as basic research, functional research, clinical research, and industrialization, necessitating robust research and development capabilities. Advanced technologies like genomics, transcriptomics, and proteomics should be employed to analyze the molecular mechanisms behind strains’ environmental tolerance, intestinal adaptation, and probiotic function (140). Nevertheless, it is crucial to note that the benefits of probiotics are contingent upon the specific strain. Consequently, each strain must undergo a time-consuming development process, presenting challenges to the widespread implementation of probiotics. Fortunately, with the continuous emergence and advancement of new technologies, the comprehensive study of probiotics is expected to become less daunting.

A number of reports have demonstrated the various effects of probiotics on digestive tract health, immune health, and metabolic health (140). However, several issues arise. For instance, researchers have persistently faced the challenge of insufficient knowledge regarding probiotic strains and dosage. Due to the availability of probiotics without a prescription, many individuals begin or discontinue their usage without consulting medical professionals or seeking specialized advice (1). Enterprises often exaggerate the efficacy of probiotics when marketing them, leading to consumers experiencing different outcomes after consumption, thereby hindering the overall development of the probiotics industry. Furthermore, although research on probiotics is rapidly expanding, the availability of concrete evidence elucidating mechanisms, particularly the key substances and the reasons for variations between strains remains limited. Undoubtedly, this presents the most critical and difficult obstacle in probiotic research and development. We anticipate that this challenge will be overcome soon, paving the way for more possibilities in probiotic applications.

## 6 Conclusion and future perspectives

An important feature of probiotics is their ability to stabilize gut microbiota homeostasis and regulate immune responses, which has led to an increase in their application among patients worldwide. While traditional treatment for RA plays a fundamental role in its management and is typically a lifelong treatment, the long-term accumulation of multiple drugs often leads to adverse effects for patients. Consequently, researchers have been investigating a new therapy that targets intestinal bacteria through the use of probiotics in order to prevent or treat RA. As discussed in this review, the gut microbiota is closely associated with the development and progression of RA. Therefore, it is crucial to identify the causal relationship between the human microbiome and the pathogenesis of RA, as this knowledge is essential for improving treatment outcomes and restoring the health of patients. The manipulability of the gut microbiota makes it possible to locally or systematically manipulate the abundance of specific gut microbiota associated with diseases through interventions, potentially changing the treatment landscape for patients with RA. If proven feasible, this approach could lead to the development of entirely new prevention or treatment strategies in the clinical field. Furthermore, animal and clinical trials have demonstrated that probiotics supplementation ameliorates the symptoms of RA by regulating dysbiosis in the gut microbiota and modulating immune responses. Despite the various challenges that remain in probiotic research, such as determining targets, pathways, and mechanisms, the numerous benefits they offer to the human body continue to drive researchers in their quest for further exploration.

## Author contributions

YY: Writing – review & editing. QH: Project administration, Writing – review & editing. XZ: Resources, Supervision, Writing – review & editing. ZL: Funding acquisition, Methodology, Supervision, Writing – review & editing.
